# Explaining the correlations among properties of mammalian promoters

**DOI:** 10.1093/nar/gku115

**Published:** 2014-03-27

**Authors:** Martin C. Frith, the FANTOM consortium

**Affiliations:** Computational Biology Research Center, AIST, 2-4-7 Aomi, Koto-ku, Tokyo 135-0064, Japan

## Abstract

Proximal promoters are fundamental genomic elements for gene expression. They vary in terms of GC percentage, CpG abundance, presence of TATA signal, evolutionary conservation, chromosomal spread of transcription start sites and breadth of expression across cell types. These properties are correlated, and it has been suggested that there are two classes of promoters: one class with high CpG, widely spread transcription start sites and broad expression, and another with TATA signals, narrow spread and restricted expression. However, it has been unclear why these properties are correlated in this way. We reexamined these features using the deep FANTOM5 CAGE data from hundreds of cell types. First, we point out subtle but important biases in previous definitions of promoters and of expression breadth. Second, we show that most promoters are rather nonspecifically expressed across many cell types. Third, promoters’ expression breadth is independent of maximum expression level, and therefore correlates with average expression level. Fourth, the data show a more complex picture than two classes, with a network of direct and indirect correlations among promoter properties. By tentatively distinguishing the direct from the indirect correlations, we reveal simple explanations for them.

## INTRODUCTION

### CAGE data and the nature of transcription initiation

CAGE (Cap Analysis of Gene Expression) is a powerful method for profiling transcription start sites (TSSs). In this method, large numbers of short sequence tags are obtained from the 5′-ends of capped RNA molecules. [short RNAs <100 bases are excluded by AMPure purification (1)]. These tags are then aligned to a reference genome sequence, and they indicate both the location of TSSs and the expression level, i.e. the proportion of RNA molecules starting at each site (2).

**Figure 1.**
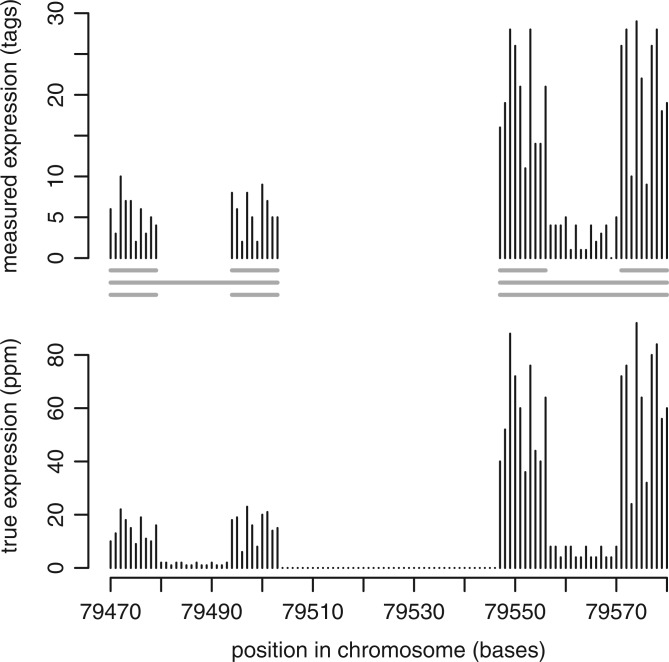
A fictional example of transcription initiation and CAGE tags. The lower graph shows the true proportions, in parts per million, of capped RNAs starting at each base on one strand of a short chromosomal segment. The upper graph shows the number of CAGE tags starting at each base. The horizontal gray lines show some ways of clustering the CAGE tags into four clusters, two clusters or three clusters.

It is important to remember that CAGE tags are samples from the RNA population. For example, in a pineal gland, one TSS might have a true expression level of 7.62 parts per million (ppm), meaning that 7.62 ppm of all the capped RNAs in that pineal gland at that moment were transcribed from that TSS. However, if we obtain a million CAGE tags, then by chance, 6 (or 10) of them might match that TSS. This relationship between population and sample is illustrated in Figure 1.

In the classic textbook view, each gene has one promoter (or a few alternative promoters), and each promoter has one TSS at exactly one nucleotide. CAGE and other data have revealed that the reality is much more messy (Figure 1): transcription can initiate at many alternative nucleotides (2–4).

In some promoters, transcription starts are spread over a wide span of nucleotides, and in others, they are concentrated in a few nucleotides. Previous studies have suggested that TSS spread correlates with CpG islands (CGIs), non–cell-specific expression and other properties (2,5–8). In these studies, the raw CAGE data (e.g. upper graph in Figure 1) was first ‘clustered’, with the idea that each cluster corresponds to a promoter. Then, each cluster’s degree of spread could be measured.

The problem is that there are different ways to cluster CAGE tags, and it is not clear which is correct. In fact, it may not even be falsifiable. For example, in Figure 1, it could be argued that there are four tight clusters, or two loose clusters. In other words, there is some arbitrariness in defining discrete promoters, just as there is arbitrariness in defining discrete mountains in a rugged landscape.

The simplest clustering method is ‘distance-based clustering’, which just links CAGE start sites that are within (say) 20 bases of each other. Previous studies have often used ‘tag overlap clustering’, which links tags that overlap after alignment to the genome. Because the tags in these earlier studies had length 20 or 21, this is almost the same as distance-based clustering. These approaches are intuitively flawed because they produce wider clusters when there are more tags. For example, in Figure 1, they may produce three clusters, even though the true expression of the left-hand double-peak has the same shape as the right-hand double-peak. This introduces a spurious correlation between expression level and TSS spread.

An alternative clustering method that avoids this flaw is ‘density-based clustering’ (9), and there are also methods that consider the similarity of each TSS’s expression profile across cell types (10,34). However, we lack certainty that any of these clustering methods avoids all subjectivity and bias. In the present study, we attempt to measure TSS spread objectively, by not using any specific clustering.

### Expression specificity

Some promoters are mainly expressed in one or a few cell types, and others are broadly expressed across many cell types. It is often desired to quantify a promoter’s expression specificity using a single number. This is another case where there are multiple ways to do it, and no one way is obviously best (11).

For example, suppose we have 100 cell types:

- Promoter A is expressed at 100 ppm in one cell type and 0 ppm in the others,
- B is expressed at 50 ppm in one and 20 ppm in the others,
- C is expressed at 30 ppm in 50 cell types and 0 ppm in the others and
- D is expressed at 10 ppm in all cell types.

Clearly A is the most and D the least specific, but opinions can differ about B and C.

This problem of measuring diversity has long been faced by ecologists and economists, who have proposed various diversity indices, such as Shannon entropy, Simpson index and Gini coefficient (11) (http://en.wikipedia.org/wiki/Diversity_index). More recently, Shannon entropy has been used to quantify gene expression specificity (12).

Another problem is that we need to estimate expression specificity from limited samples of CAGE tags. Unfortunately, Shannon entropy and other diversity indices have a systematic bias: when calculated from limited samples, the estimated specificity tends to be higher than the true specificity (13–16).

A different problem is that it can be unclear whether two cell types are different. For example, suppose we measure a promoter’s expression in aortic smooth muscle, bladder smooth muscle, bronchial smooth muscle, prostate smooth muscle, hepatocytes and macrophages. If we treat all these cell types as equally different, it seems we may get a biased picture of expression specificity. On the other hand, it has been suggested that smooth muscle in particular (in addition to fibroblasts and neurons) may represent an ‘uncalculated diversity of cell types’ (17). All existing expression atlases, including FANTOM5, are biased toward easily accessible cell types (such as muscle) and lack others (such as rare embryonic cell types).

### CpG islands

Mammalian genomes are strongly depleted in CG dinucleotides (relative to the abundance of C and G), and CGIs are short segments that are less depleted in CG. The likely reason for CG depletion is that CG dinucleotides are usually methylated on the cytosine, and methylcytosine has a high mutation rate. CGIs often overlap proximal promoters, and promoter CG methylation is associated with transcriptional silencing. This leads to a simple explanation of CGIs: they reflect promoters that are active and thus unmethylated in germ line cells. (Recall that germ line cells are those whose DNA and mutations can pass to offspring.) In particular, ‘the pattern of CGIs in the genome should reflect a weighted average of methylation patterns in the germ line for which the weight is proportional to the time spent in the particular methylation state’ (18).

This is a beautifully parsimonious explanation of CGIs, which does not require ascribing any function to them: they are merely passive consequences of methylation and mutation patterns in germ cells. (Of course, this does not rule out that CGIs may occasionally contribute motifs that become exapted to function as, say, Sp1 binding sites.)

An alternative theory is that CGIs are functional elements. It has been suggested that they are nucleosome-destabilizing elements (19), and that they influence chromatin modification state (20). If CGIs are functional, they ought to experience evolutionary selection (19,20). A recent study examined this question using mathematical models of sequence evolution, concluding that hypomethylation, and not selection, largely explains CGIs (21). All in all, there is confusion regarding why CGIs exist.

### The FANTOM5 data

The FANTOM5 phase 1 data has CAGE tags from 517 human samples (after pooling replicates). These include ‘primary cells’ (e.g. amniotic membrane cells, salivary acinar cells, tenocytes), ‘tissues’ (e.g. achilles tendon, adrenal gland, amygdala) and ‘cell lines’ (e.g. glioma cell line GI-1, teratocarcinoma cell line PA-1). The primary cells will not perfectly represent pure *in vivo* cell types because of the imperfect procedures used to obtain and handle them. The tissues and cell lines are useful because they include cell types and promoters missing from the primary cell collection. In this study, we used all the samples on an equal footing, except where stated otherwise.

There are also 290 mouse samples, with more tissues than the human samples, but fewer primary cells and much fewer cell lines. Twelve human samples (all cell lines) and no mouse samples are germ cells, according to the FANTOM5 ontology.

This work is part of the FANTOM5 project. Data downloads, genomic tools and copublished manuscripts are summarized here http://fantom.gsc.riken.jp/5/.

## MATERIALS AND METHODS

We conservatively chose to use only CAGE tags near starts of RNAs in the RefSeq database (22,23). There are many more CAGE clusters than RefSeq starts, but it is unclear how many of them reflect promoters and how many reflect something else (e.g. artifacts of the CAGE method). There is a risk that RefSeq starts underrepresent promoters with low and/or highly cell-specific expression. Another danger is that RefSeq RNAs are based on RNA sequence data, which is not necessarily more reliable than CAGE and may suffer from the same artifacts. However, to make progress, we provisionally accept that these adequately represent true promoters.

Our first thought was to use CAGE tags starting up to 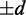 bases from each RefSeq start (*d* = 50). The problem is that RefSeq starts are sometimes slightly upstream of the main CAGE peak (perhaps because RefSeq uses the most-upstream transcript evidence). Therefore, we (i) found locally maximal CAGE start sites that have more tags than any other site up to 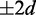 either side, (ii) discarded locally maximal sites more than 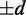 bases from a RefSeq start and (iii) used CAGE tags starting up to 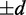 bases from each locally maximal site. This gave us a set of 17 039 ‘promoters’.

To ensure our conclusions are robust, we also tried *d* = 20, *d* = 100 and *d* = 200, as well as mouse data with *d* = 50 (Supplementary Figures S1–S8). The conclusions do not change.

### Sample ontology

To identify and pool replicate CAGE samples, we looked for samples that have identical ‘is_a’ terms in the FANTOM5 ontology.

### Entropy

The expression level of promoter *g* in sample *t* is 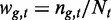, where 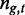 is the number of CAGE tags in that promoter and sample, and *N_t_* is the number of mapped tags in the sample. To calculate entropy with pseudocounts, we used this alternative formula: 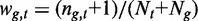, where *N_g_* is the number of promoters (17 039). The entropy of promoter *g* is 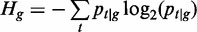, where 
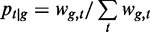
.

### Simulated promoters

Each simulated promoter is uniformly expressed in all cell types, 50% of cell types (chosen at random per promoter), 25% of cell types or 10% of cell types. Each simulated promoter has the same total tag count as the corresponding real promoter. Each tag was randomly assigned to a cell type, with probability proportional to the number of mapped tags for that cell type.

### CpG and %G+C

We counted CpGs and %G+C in the length-201 sequence centered on each promoter.

### TATA motifs

We found the highest-scoring TATA match within the length-(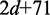) sequence centered on each promoter, on the coding strand. The score for base *x* at position *k* in the motif is 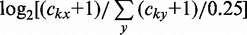, where *c_kx_* is the count for base *x* at position *k* in JASPAR matrix MA0108.2 (24).

### Percentage identity versus mouse

We measured the percentage identity for the length-100 sequence immediately upstream of each promoter’s central base. Percentage identity here means the percentage of human bases that are aligned to an identical mouse base, in the hg19 vsMm9 axtNet files from the University of California, Santa Cruz (UCSC) genome database (23).

### Miscellaneous

We used RefSeq annotations from FANTOM5’s 1 January 2012 snapshot of the UCSC genome database. We calculated correlation coefficients and their *P*-values using the *R* function cor.test, and partial correlations using the *R* package ppcor (25).

## RESULTS

### Sampling depth confounds expression specificity

**Figure 2.**
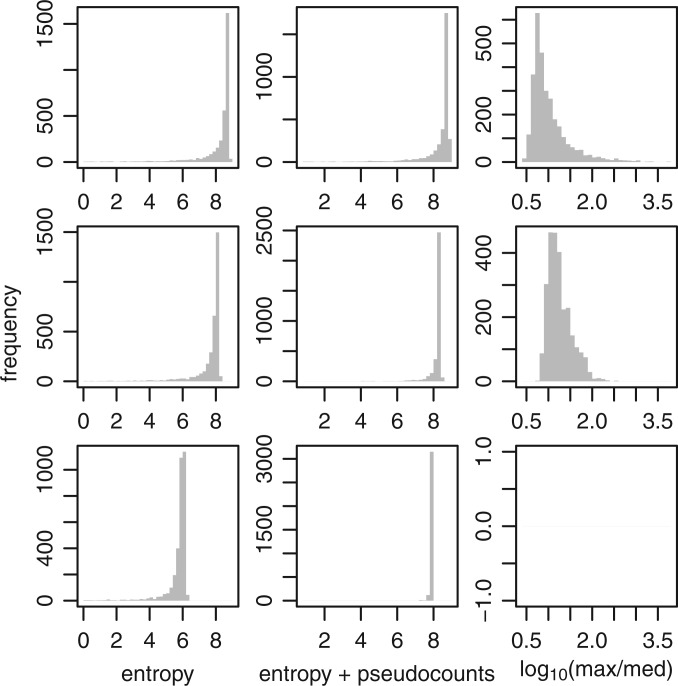
Sampling depth confounds three measures of expression specificity. We took the 3287 promoters with at least 10^5^ tags, and from each promoter, we randomly sampled (with replacement) 10^4^ tags (upper row), 1000 tags (middle row) or 100 tags (lower row). The lower-right panel is empty because with 100 tags and >200 cell types the median expression is necessarily 0.

We attempted to quantify each promoter’s expression specificity, using entropy, which varies from 0 for promoters expressed in a single sample to 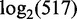 for promoters with perfectly uniform expression (12). As mentioned above, entropy estimates from limited samples have a systematic bias, but it is not obvious whether this will be significant or negligible in our case. To examine this, we performed a simulation: we took the 3287 promoters with 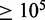 tags, randomly sampled 10^2^, 10^3^ or 10^4^ tags from each promoter and calculated the entropies. The entropy tends to decrease as the sample size decreases (Figure 2, left column). The difference between 10^3^ and 10^4^ looks small, but it is large relative to the tightness of the distribution.

The underlying problem is that, if we get a limited sample of tags from a promoter with fairly uniform expression across cell types, the expression looks more spiky than it really is. One possible solution is to use pseudocounts, i.e. add one to each promoter’s tag count in each sample (12). In our simulation, pseudocounts did not solve the problem: the entropy still tends to decrease with decreasing sample size (Figure 2, middle column). Again, the decrease in entropy looks small, but it is large relative to the tightness of the distribution. Moreover, with 10^2^ tags there are more pseudocounts than real counts, so the specificity estimates are not believable.

An alternative measure of specificity is a promoter’s maximum expression level in any sample divided by its median expression level (34). This varies from one for uniform expression, to large values for specific expression. In our simulation, this also exhibits a bias: the apparent specificity increases with decreasing sample size, even though the real specificity does not change (Figure 2, right column).

In summary, it is not straightforward to quantify expression specificity in a way that does not correlate artifactually with sampling depth. This artifact matters because tag count correlates with other properties, such as average expression level and CpG content. So, for example, it might introduce a spurious correlation between CGIs and apparent breadth of expression across cell types. An important message is that future studies need to be careful when assessing expression specificity. In this study, we solve the problem by using the entropy of a random sample of 100 tags from each promoter, thus fixing the sampling depth to a constant.

### TSS spread

**Figure 3.**
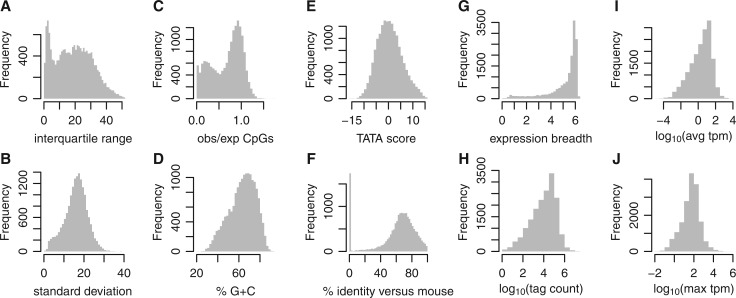
Histograms of 10 promoter properties: chromosomal spread of TSSs measured by IQR (**A**) and SD (**B**), observed/expected CpGs (**C**), G+C percentage (**D**), score of TATA motif match (**E**), evolutionary conservation (**F**), breadth of expression across cell types (**G**), number of CAGE tags (**H**), average expression level (**I**), and maximum expression level (**J**). Expression breadth is the entropy (in bits) of a random sample of 100 tags from a promoter.

We quantified each promoter’s TSS spread using two standard measures of spread: interquartile range (IQR) and standard deviation (SD). That is, for every tag in the promoter, we noted its start coordinate, and calculated the IQR and SD of these coordinates. IQR shows a bimodal distribution (Figure 3A): there is one class of promoters with narrow spread (IQR < 7, peaking around 2), and another with wide spread (IQR > 7, peaking around 20). This agrees with previous findings (2,5). It is a bit disturbing that SD does not show clear bimodality (Figure 3B), but the distribution has a bulge at low SD that could be interpreted as the narrow-TSS-spread class.

### Expression specificity

**Figure 4.**
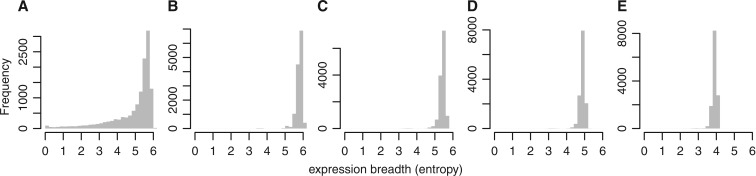
Histograms of promoters’ expression breadth across cell types, using FANTOM5 ‘primary cells’ only. Expression breadth is the entropy (in bits) of a random sample of 100 tags from a promoter. (**A**) Real data. (**B**) Simulated data, where each promoter has uniform expression across all cell types. (**C**) Simulated data, where each promoter has uniform expression across 50% of the cell types (chosen at random) and 0 in the other 50%. (**D**) Simulated data: uniform expression across 25% of cell types and 0 in the others. (**E**) Simulated data: uniform expression across 10% of cell types and 0 in the others.

Most promoters show broad expression across many cell and tissue samples (Figure 3G). It might be objected that this is due to the tissue samples being mixtures of cell types. To address this concern, we also measured expression breadth using the ‘primary cell’ samples only. This hardly changes the picture (Figure 4A), although there is a small peak of promoters with highly cell-specific expression (entropy close to 0).

To calibrate our entropy scale, we show results for four simulated data sets: promoters with uniform expression in 100, 50, 25 and 10% of the cell types (Figure 4B–E). This confirms that most real promoters are broadly expressed across many cell types, though not uniformly expressed in all cells. For instance, the real promoters have a median entropy of 5.41 (Figure 4A), similar to the simulated promoters with uniform expression in 50% of cell types (Figure 4C). Only 18% of the real promoters have entropy <4 (similar to the simulated promoters with uniform expression in 10% of cell types), which might be considered somewhat specific. Promoters expressed in just one cell type have entropy = 0, and there are only 48 of those.

Curiously, 31 of the 48 cell-specific promoters are active in hepatocytes. They include promoters for *ADH1A* (alcohol dehydrogenase 1A), *ALDOB* (aldolase B), coagulation factors *F9* and *F12* and several cytochrome P450 genes.

That most promoters are broadly expressed was also shown using a richness index (34) (Supplementary Note 3). Richness is simply the number of samples in which we would expect to find one or more tags for a given promoter, if that promoter had (say) 100 tags in total (11). For most promoters, if we had 10 tags in total, they would likely be distributed among 9 or 10 different samples. Thus, most promoters are expressed almost as uniformly across samples as they could possibly be.

### Other promoter properties

We also measured the following:

- %G+C.
- Observed/expected CpGs, i.e. the observed number of CG dinucleotides divided by the number expected from %G+C.
- Score of strongest TATA motif match. We found the best match in every promoter, no matter how weak, so this will be a mixture of real signals and random matches.
- Percentage sequence identity versus mouse.
- Tag count.
- Average expression level (tags per million, tpm).
- Maximum expression level (tpm).

Their distributions are not too surprising (Figure 3). CpG richness is bimodally distributed (18). Some promoters have 0% identity versus mouse, either because they are in large evolutionary insertions or deletions, or because the UCSC genome alignments failed to align them.

### Expression specificity is independent of maximum expression level

It is not obvious how expression specificity relates to expression level. One hypothesis is that promoters’ expression specificity should be independent of their average expression level across cell types. This fits a picture where each promoter has a default expression level, which can be upregulated in some cell types and downregulated in others. The more the regulation, the greater is the specificity. In this case, we would expect specificity to correlate with maximum expression level.

Another hypothesis is that promoters’ specificity should be independent of their maximum expression level. This fits a picture where each promoter has a maximum expression level, which is near-fully achieved in more or fewer cell types. The fewer the cell types where the promoter is near-fully on, the greater is the specificity. In this case, we would expect specificity to anticorrelate with average expression level.

**Figure 5.**
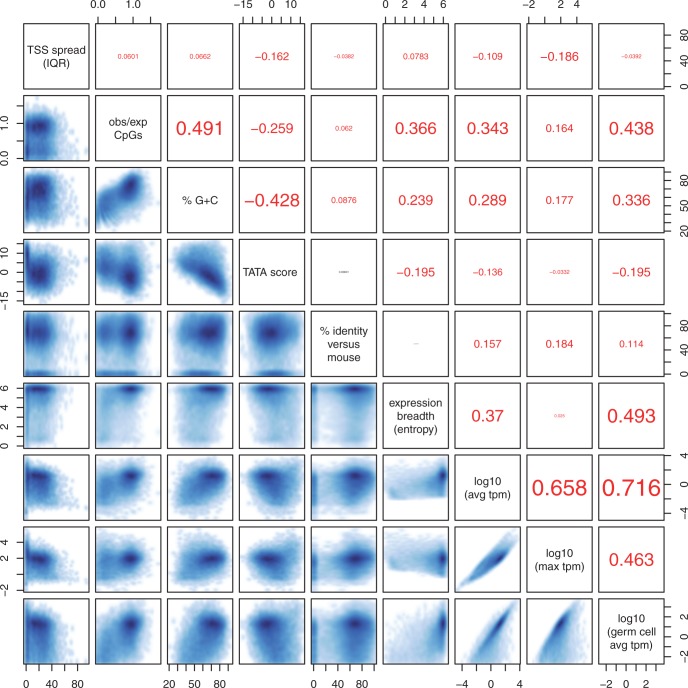
Pairwise correlations among nine promoter properties. The numbers in the upper-right boxes are Kendall rank-order correlation coefficients (Txy). Statistically significant (two-sided 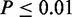) coefficients are in red, and nonsignificant ones are in black.

In fact, expression breadth (entropy of a sample of 100 tags) is almost independent of maximum expression level, and correlates with average expression level (Figure 5). This fits the second hypothesis.

### Correlations

We examined pair-wise correlations among nine promoter properties (Figure 5). The picture is complex: nearly all pairs are significantly correlated or anticorrelated. Some of these associations could be indirect. For example, there is a correlation between expression breadth and %G+C, but there are stronger correlations between each of these and germ cell expression. So the correlation between expression breadth and %G+C might be merely an indirect consequence of the stronger correlations.

**Figure 6.**
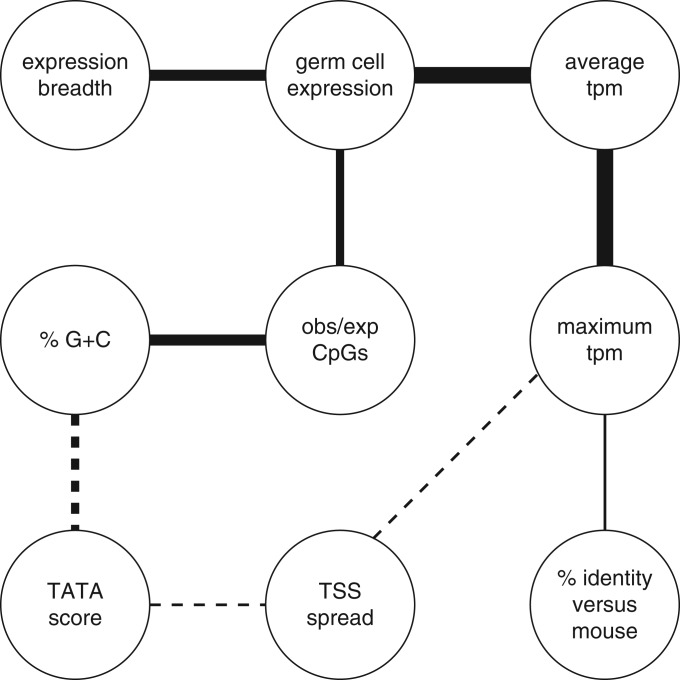
A model for correlations among several promoter properties. Solid lines represent positive correlations and dashed lines represent negative correlations. Line widths are proportional to correlation strength.

We attempted to distinguish direct from indirect associations, by discarding all associations that can potentially be explained by stronger ones. This leads to an interesting model that explains all the correlations (Figure 6). Strikingly, almost all of the putatively direct associations have simple explanations:

#### Average tpm and germ cell average tpm

This is the strongest correlation in Figure 5. It is not surprising because average tpm includes germ cell tpm. (Therefore, average tpm must be 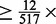
germ cell average tpm.)

#### Average tpm and maximum tpm

This is the second-strongest correlation. It is also not surprising because average tpm must lie between maximum tpm and (maximum tpm)/517. Accordingly, the scatterplot lies in a diagonal band between these bounds (Figure 5).

#### Germ cell expression and expression breadth

In fact, expression breadth correlates not only with germ cell expression, it correlates with expression in any given cell type. For example, it correlates with expression in kidney (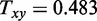) and lung (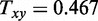) samples. This can be explained by the following two observations: (i) expression breadth is independent of maximum expression level; (ii) the greater a promoter’s breadth of expression, the more likely it is to be expressed near its maximum level in any given cell type.

#### Observed/expected CpGs and %G+C

This correlation can be explained by the methylation/mutation mechanism behind CpG depletion. This mechanism strongly depletes CG dinucleotides, and weakly depletes C and G mononucleotides (26). Because this mechanism acts more strongly in some promoters than others, it causes a correlation between %G+C and observed/expected CpGs. Another possible explanation is GC-biased gene conversion during recombination. This phenomenon increases %G+C in recombination-prone regions of the genome (27), whereas DNA methylation and transcriptional silencing suppress recombination (28).

#### Germ cell expression and observed/expected CpGs

This correlation fits the simple explanation of CGIs that they are due to nonmethylation of active promoters in germ line cells (29).

#### %G+C and TATA score

There are perhaps two reasons for this anticorrelation because our TATA matches include both real signals and random matches. The first is trivial: random TATA matches are likely to be weaker in GC-rich sequences. The second is less trivial: we suggest that real TATA signals are evolutionarily favored in GC-poor sequences. The assumption is that many promoters can function equally well with either a real TATA signal, or alternative non-TATA promoter signals. For such promoters, if mutational patterns tend to enrich for A and T, evolution is more likely to produce real TATA signals.

#### TSS spread and maximum tpm

TSS spread (IQR) is only weakly associated with the other properties (Figure 5). The strongest association is anticorrelation with maximum tpm. We cannot explain this as easily as the other associations. One possible explanation is that, when the maximum tpm is low, the CAGE tags might include a proportionally larger amount of diffuse ‘noise’.

#### Evolutionary conservation and maximum tpm

Evolutionary conservation (Percentage identity versus mouse) is only weakly associated with the other properties (Figure 5). The strongest correlation is with maximum tpm. This can be explained by the plausible hypothesis that promoters with higher maximum expression tend (slightly) to be more important and conserved in mammalian biology.

#### TSS spread and TATA score

TSS spread (IQR) is weakly anticorrelated with TATA score (Figure 5). This makes sense because the TATA motif is one of several signals that influence TSS position (9).

### Partial correlations

We have suggested that many of the associations in Figure 5 are indirect, i.e. merely consequences of other direct associations. This can be tested using partial correlation. The partial correlation coefficient 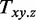 indicates the correlation between *x* and *y* after eliminating the influence of *z*. Thus, if *x* and *y* are indirectly correlated via *z*, 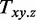 will be near-zero and nonsignificant.

Unfortunately, there is a practical limitation. If *x* and *y* are indirectly correlated via *z*, but our measurements of *z* are not perfect, then 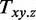 can be highly significant (30).

Table 1 shows some examples. It suggests that the anticorrelation between TATA score and observed/expected CpGs is mostly, but not entirely, explained by the associations of both with %G+C. One interpretation of this is that %G+C is an imperfect proxy for the mutational patterns that influence TATA evolution.

**Table 1.** Partial correlations

*x* ∼ *y*	z	*T_xy_* (*p*)	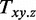 (*p*)
CpG ∼ TATA	%G+C	–0.259 (0)	–0.0619 (9.01e–34)
CpG ∼ %G+C	TATA	0.491 (0)	0.435 (0)
TATA ∼ %G+C	CpG	–0.428 (0)	–0.358 (0)
CpG ∼ ExB	GCE	0.366 (0)	0.208 (0)
CpG ∼ GCE	ExB	0.407 (0)	0.28 (0)
ExB ∼ GCE	CpG	0.493 (0)	0.404 (0)

*T_xy__,_* Kendall rank-order correlation coefficient; 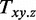, Kendall partial rank-order correlation coefficient; *p,* two-sided *P*-value; ExB, expression breadth (entropy of a random sample of 100 tags); GCE, germ cell expression (average tpm). (The correlation between CpG and GCE differs from that in Figure 5 because here we omitted 7.6% of promoters with <100 tags.)

On the other hand, the correlation between expression breadth and observed/expected CpGs seems to be only partly explained by the correlation of both with germ cell expression (Table 1). However, our measurement of germ cell expression is extremely imperfect because our germ cell data comes entirely from cell lines. So it remains possible that the correlation between expression breadth and observed/expected CpGs is entirely indirect via germ line activity. In future, it will be informative to get CAGE data from various types of primary germ line cell, given their importance for sequence evolution.

### A closer look at TATA motifs

There is more than one way to find TATA motifs. So far, we have looked for good matches to a TATA model relative to a uniform background model. It is also possible to use a per-promoter background model, which uses the base frequencies of each promoter. With a per-promoter background model, the score of a given TATA sequence is reduced if the promoter is AT-rich (because TATA matches are less surprising), and increased if the promoter is AT-poor. So it is not obvious whether our conclusions will be the same.

**Figure 7.**
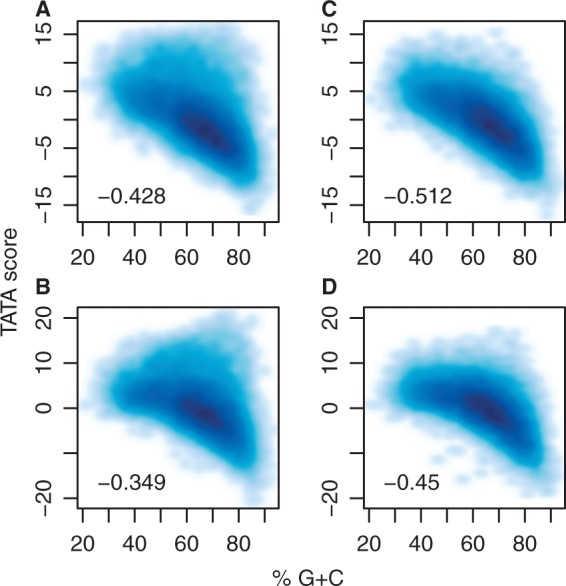
Correlation between TATA motif and G+C composition. (**A**) Real promoters, uniform background model. (**B**) Real promoters, per-promoter background model. (**C**) Shuffled promoters, uniform background model. (**D**) Shuffled promoters, per-promoter background model. The inset numbers are Kendall rank-order correlation coefficients (*T_xy_*).

In fact, our conclusions do not change when we use a per-promoter background model. The anticorrelation between TATA score and %G+C is reduced, but not greatly so (Figure 7). This anticorrelation is not surprising because we observe an anticorrelation if we randomly shuffle each promoter sequence (Figure 7).

### Promoter classes

It has been suggested that there are two (or perhaps three) classes of promoter: one class with wide TSS spread, often CGIs, fast evolution and broad expression across cell types; and another with narrow TSS spread, low CpG content, often TATA signals, slow evolution and cell-restricted expression (2,7,8). On one hand, the correlations in Figure 5 do not suggest clear-cut classes. On the other hand, Figure 3 suggests two CpG classes (high and low), and two TSS spread classes (wide and narrow). So the question arises of whether the high-CpG class *is* the wide-spread class.

**Figure 8.**
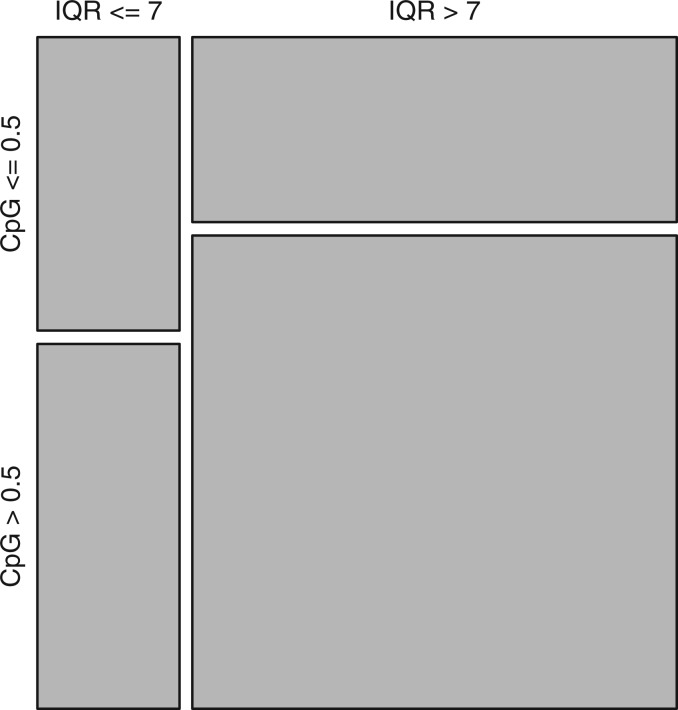
Mosaic plot of high/low CpG versus high/low chromosomal spread of TSSs. The area of each rectangle is proportional to the number of promoters in each category.

To visualize this intuitively, we show a mosaic plot reflecting the numbers of promoters in the four possible combinations of high/low CpG with wide/narrow TSS spread (Figure 8). This confirms there is a correlation, but in our opinion the correlation is not strong enough to say that the high-CpG class is the wide-spread class.

We still lack explanations for why CpG rate and TSS spread are bimodal. Presumably, the CpG rates indicate that many promoters are either usually (housekeeping) or rarely active in the germ line, and fewer are active for intermediate amounts of time.

## DISCUSSION

The first message of this study is that definition of promoters, and of their expression specificity, is partly arbitrary, and there is a danger of artifactual correlations with other promoter properties. The base reality is a rugged landscape of transcription initiation at each genomic nucleotide, and promoters—peaks in this landscape—are a partly subjective abstraction. Simple promoter definitions based on nearness of CAGE tags produce wider promoters in regions with more tags, introducing an artifactual correlation between TSS spread and expression level. This will further cause artifactual indirect correlations, e.g. between TSS spread and CGIs.

The definition of expression specificity is also partly arbitrary, and typical measures such as Shannon entropy have a bias when applied to a sample of expression counts: the measured specificity tends to decrease as the sample size decreases. This causes a spurious anticorrelation between expression level and specificity.

To understand the correlations among promoter properties, the first step must be to avoid such biases. This study has done so in crude but effective ways (fixed promoter width and sample size). A frightening point is that these biases were not obvious to us initially, and similar biases probably exist undetected in other genomic studies. This seems especially likely in large projects with many contributors and diverse results. Genomic research is perhaps especially prone to statistical artifacts (30–32).

The second message of this study is that most promoters have rather nonspecific (but not perfectly uniform) expression across many cell types. Some other studies have emphasized that most promoters have nonuniform regulated expression patterns (12,34). We do not contradict that, but we do emphasize that most promoters have broad, albeit not completely uniform, expression.

This result has implications for how cell types are determined, and how they evolved. We can rule out the idea that most cell types are determined by expressing hundreds of unique genes. (Because there are hundreds of human cell types (17), if most expressed hundreds of unique genes, there would have to be tens of thousands of cell-specific promoters, which is contradicted by Figure 4.) Actually, this is obvious when we consider that humans have many more cell types than some invertebrates, but not many more genes. It implies that cell types are determined by unique combinations of genes, or perhaps by what genes they do not express.

It has been suggested that cell types evolved by three main mechanisms: (i) segregation of functions, starting from multifunctional ancestral cell types, with loss of gene expression in descendant cells; (ii) divergence of functions, often driven by gene duplication and divergence; and (iii) acquisition of new functions, sometimes by co-option of genes from other cell types (33). Our data suggest that loss of gene expression and co-option may have been the major mechanisms.

Our third message is that promoters’ expression breadth is independent of their maximum expression level, and therefore correlates with average expression level. This may seem an obscure finding, but it is in fact fundamental, and it is not obvious a priori. In evolutionary terms, it suggests that genes became tissue-specific mainly by being downregulated, rather than upregulated, in newly evolving cell types.

Our fourth message is that the intriguing correlations between basic properties of promoters can almost all be explained simply. The heart of this explanation is that expression breadth correlates with expression level in any given class of cells, including germ cells, and germ cell expression reduces the CpG mutation rate. In addition, TATA motifs are naturally anticorrelated with %G+C and CpGs, and they reduce TSS spread. Thus we need not invoke any direct functional relationship between CGIs and expression breadth or TSS spread.

These explanations are consistent with the parsimonious theory of CGIs, that they are nonfunctional consequences of mutation patterns in the germ line. It has been shown that CGI-containing sequences have a nucleosome-destabilizing function (19). However, correlation is not necessarily causation. It is possible that some unknown sequence property causes the nucleosome destabilization, which might then lead to expression in germ cells, causing CGI evolution.

## SUPPLEMENTARY DATA

Supplementary Data are available at NAR Online.

## FUNDING

FANTOM5 was made possible by a research grant for RIKEN Omics Science Center from Ministry of Education, Culture, Sports, Science and Technology, Japan (MEXT) (to Y. H.) and a grant of the Innovative Cell Biology by Innovative Technology (Cell Innovation Program) from the MEXT, Japan, (to Y.H.). Funding for open access charge: AIST (National Institute for Advanced Industrial Science and Technology).

*Conflict of interest statement*. None declared.

## Supplementary Material

Supplementary Data
