# Supplementary material for: Explaining the correlations among properties of mammalian promoters
Source: Nucleic Acids Res. 2014 Mar 27;42(8):4823–32. doi: 10.1093/nar/gku115 (PMC4005656; doi:10.1093/nar/gku115)
Supplement: Supplementary Data [file supp_42_8_4823__index.html]

Explaining the correlations among properties of mammalian promoters — Explaining the correlations among properties of mammalian promoters — Supplementary Data 

# Explaining the correlations among properties of mammalian promoters

## Supplementary Data

files

**Files in this Data Supplement:**

- Supplementary Data - pdf file
