# Supplementary material for: Explaining the correlations among properties of mammalian promoters
Source: Nucleic Acids Res. 2014 Mar 27;42(8):4823–32. doi: 10.1093/nar/gku115 (PMC4005656; doi:10.1093/nar/gku115)
Supplement: Supplementary Data [file supp_gku115_nar-03249-z-2013-File002.pdf]

Supplement to: Explaining the correlations among properties of mammalian promoters

Martin C. Frith and the FANTOM consortium

December 12, 2013

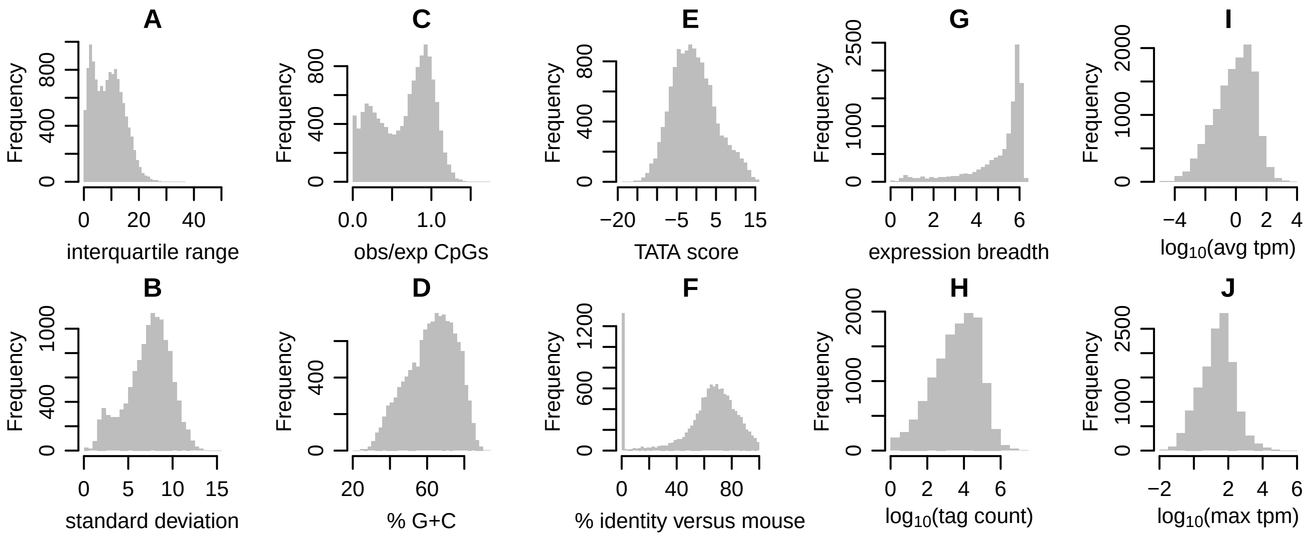

Figure 1: Histograms of ten promoter properties. This is the same as fig 3 in the main paper, but with $d = 20$.

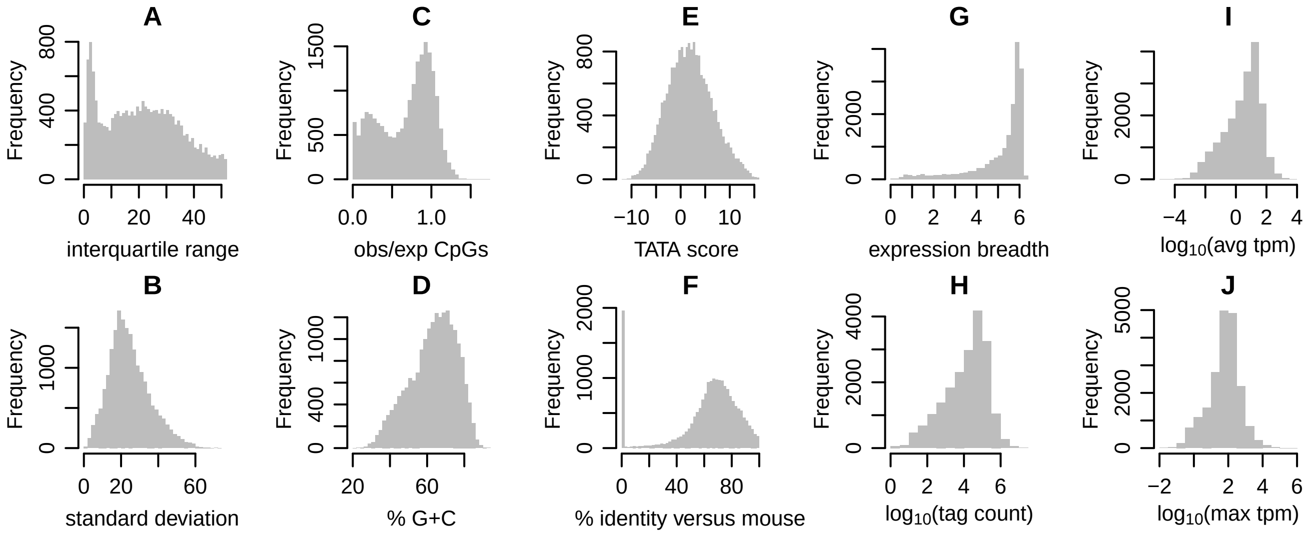

Figure 2: Histograms of ten promoter properties. This is the same as fig 3 in the main paper, but with $d = 100$.

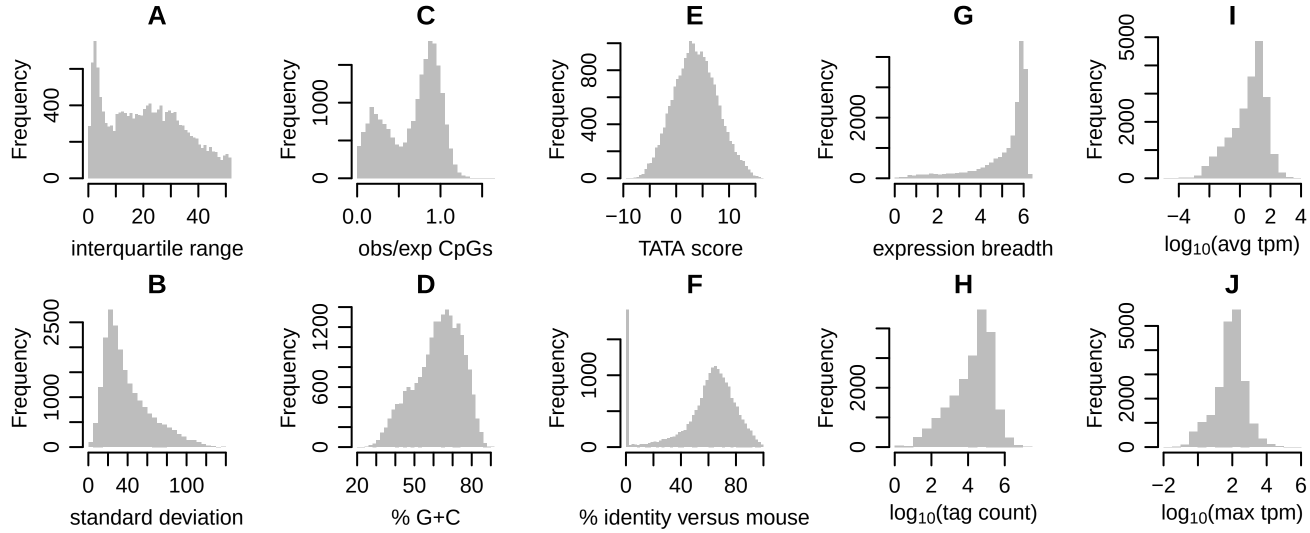

Figure 3: Histograms of ten promoter properties. This is the same as fig 3 in the main paper, but with $d = 200$.

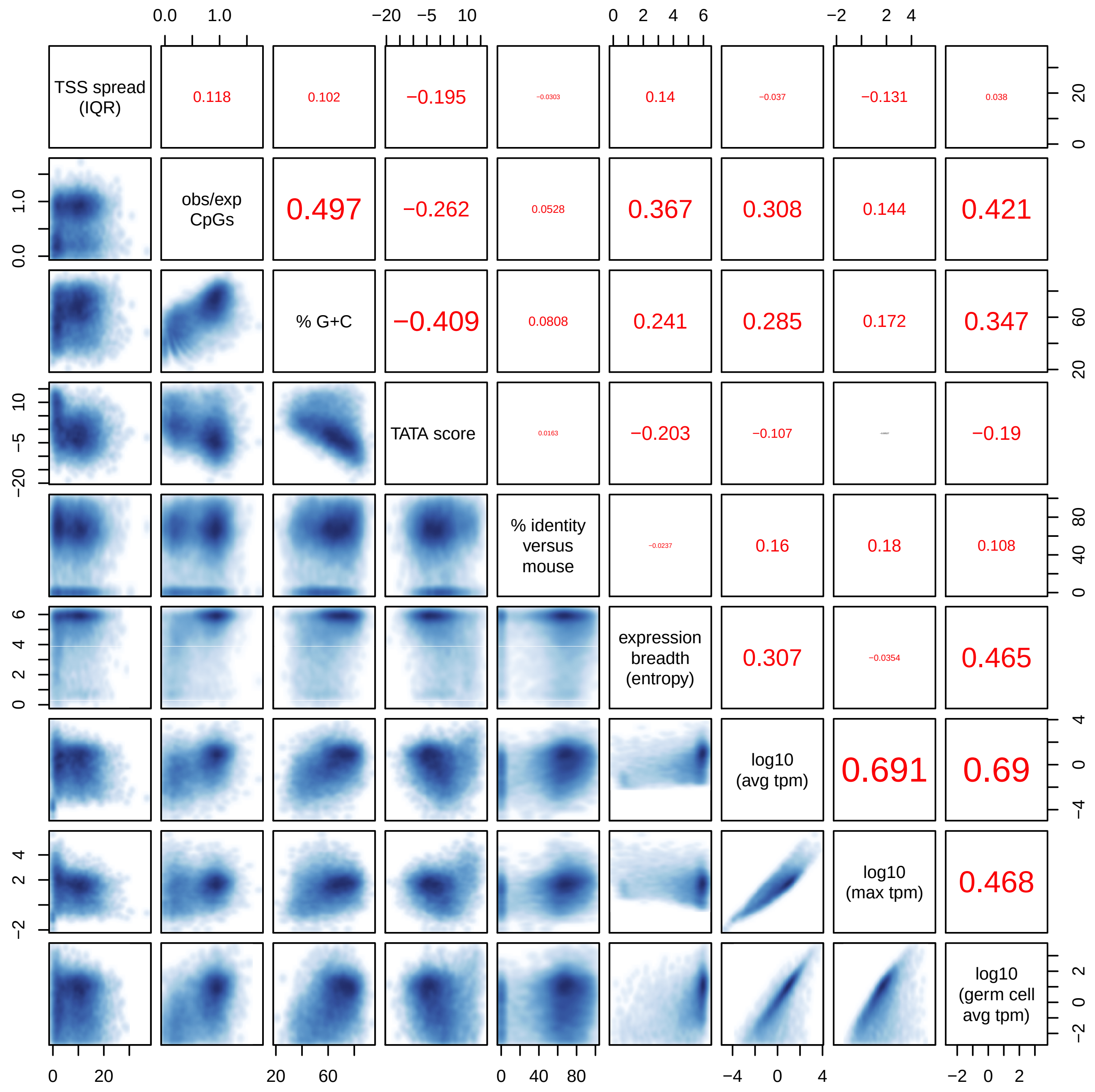

Figure 4: Pairwise correlations among nine promoter properties. This is the same as fig 5 in the main paper, but with $d = 20$.

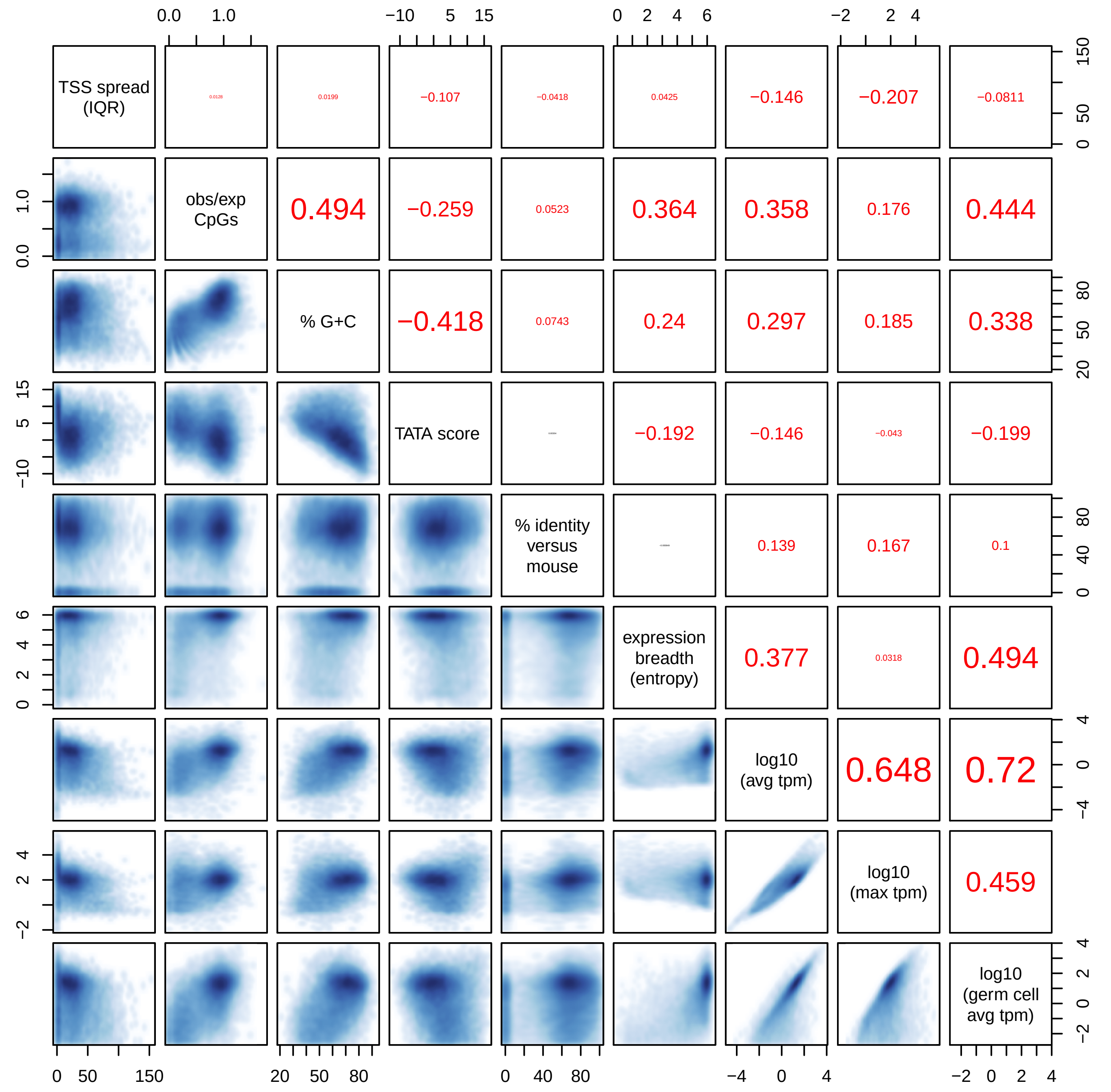

Figure 5: Pairwise correlations among nine promoter properties. This is the same as fig 5 in the main paper, but with $d = 100$.

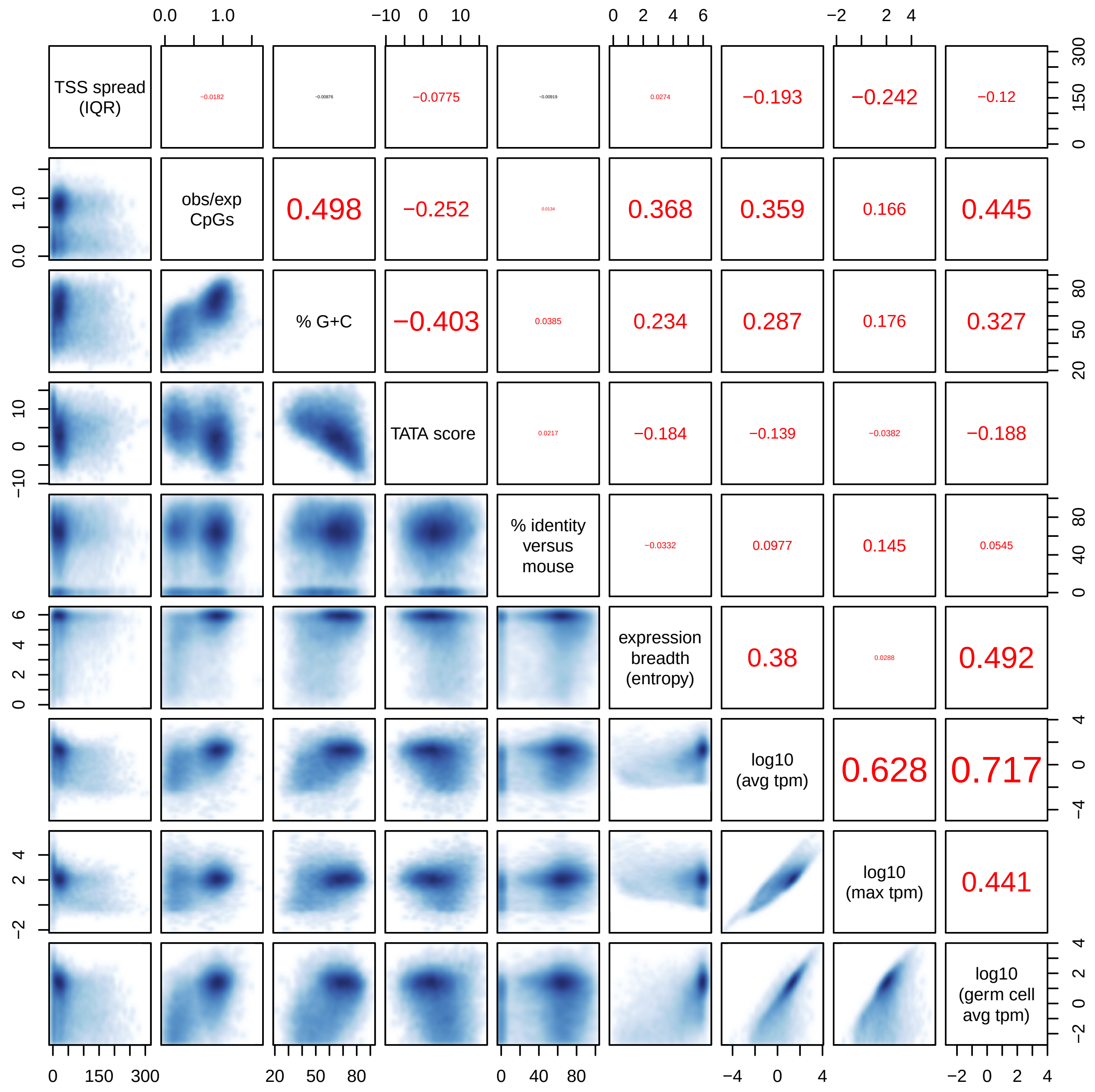

Figure 6: Pairwise correlations among nine promoter properties. This is the same as fig 5 in the main paper, but with $d = 200$.

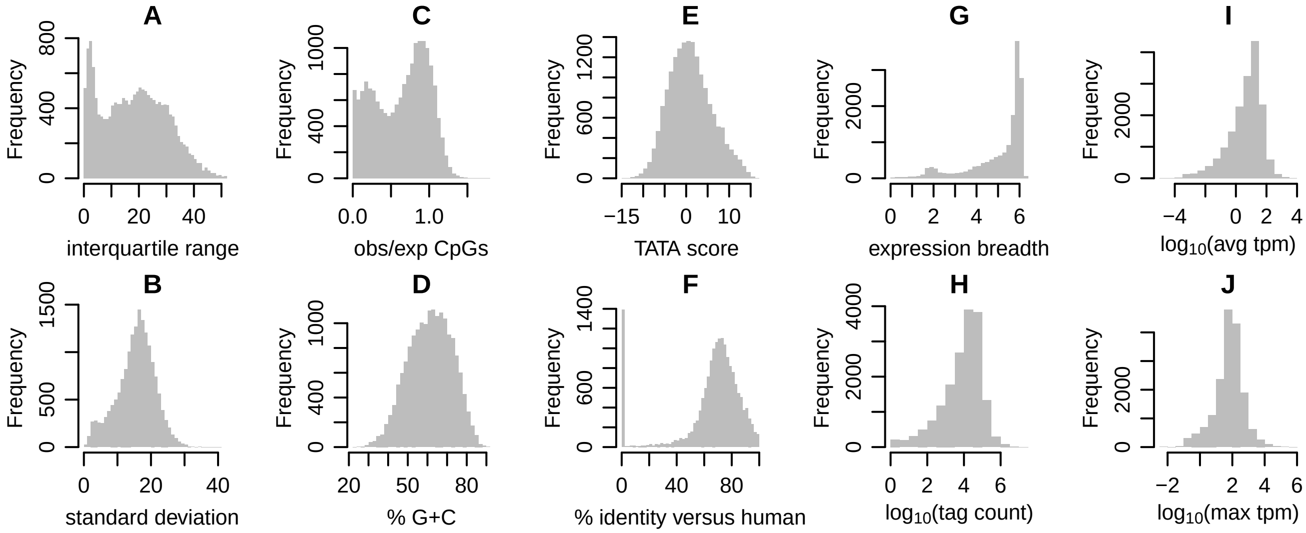

Figure 7: Histograms of ten promoter properties. This is the same as fig 3 in the main paper, using mouse instead of human data.

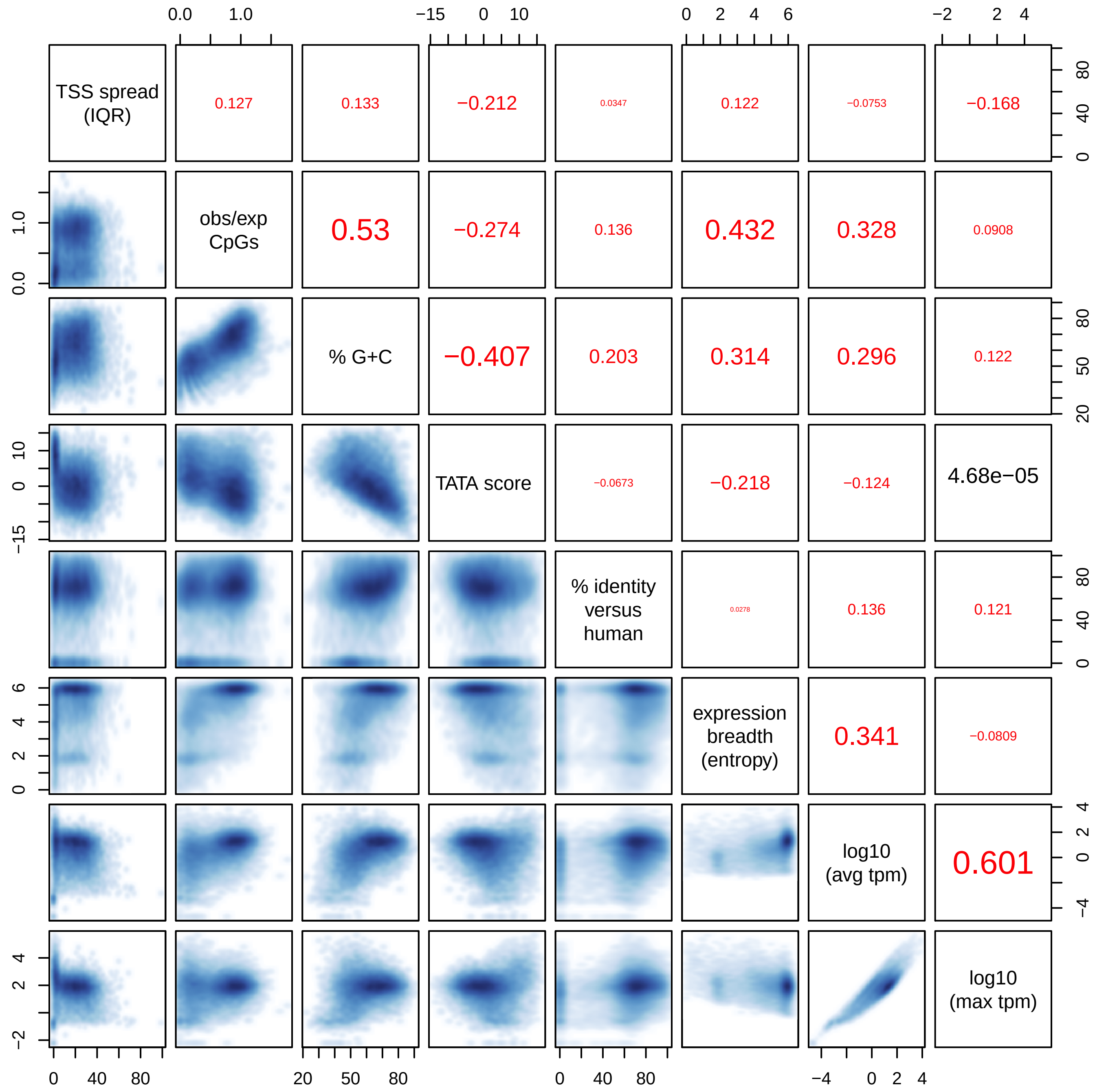

Figure 8: Pairwise correlations among eight promoter properties. This is the same as fig 5 in the main paper, using mouse instead of human data. Germ cell expression is omitted, because we lack CAGE data for mouse germ cells.
